# Editorial: Detection, characterization, and management of plant pathogens

**DOI:** 10.3389/fpls.2024.1354042

**Published:** 2024-02-13

**Authors:** Islam Hamim, Brent Sipes, Yanan Wang

**Affiliations:** ^1^Department of Plant Pathology, Faculty of Agriculture, Bangladesh Agricultural University, Mymensingh, Bangladesh; ^2^Institute of Evolutionary Ecology and Conservation Genomics, University of Ulm, Ulm, Germany; ^3^Department of Plant and Environmental Protection Sciences, University of Hawaii at Manoa, Honolulu, HI, United States; ^4^College of Plant Protection, Hebei Agricultural University, Baoding, China

**Keywords:** pathogen, agro-ecosystem, bio-diversity, genomics, management

Plant pathogens threaten agricultural sustainability and result in severe economic losses. The fast evolution of pathogens, environmental change, and international trade are the key contributors to the genesis of novel plant diseases (Ristaino et al., 2021). For plant disease management, timely and exact pathogen identification is essential. On the other side, the diversity and spread of plant pathogens can severely impede efforts to diagnose and manage pathogens (Rubio et al., 2020). Plant pathogens adopt a variety of strategies that result in host adaptability, transmission, and strain diversity (Anderson et al., 2010). A range of species, including plants, endophytes, insects, pollinators, and other pathogens, interact with plant pathogens. The effects of diseases on various plants, as well as the transmission and evolution of plant pathogens in hosts, are frequently unknown in agroecosystems and natural biosystems. We developed the Research Topic “*Detection, characterization, and management of plant pathogens*” under the section “Plant Pathogen Interactions” of the journal “Frontiers in Plant Science.” This topic was developed to enrich current knowledge of plant pathology by compiling the most recent research on plant pathogens. A better understanding of plant pathogens will help in the development of efficient plant disease control measures and assure the security of agricultural yields and the world’s food requirements in the context of climate change and the expanding global population. We were interested in collecting papers on the following topics: detection, identification, and characterization of plant pathogens; diversity, emergence, evolution, distribution, and transmission of plant pathogens; pathogenesis; and management of plant diseases in agricultural and wild systems. However, the final Research Topic was expanded to include beneficial microorganisms in agroecosystems not specified in the title in order to account for their potential role in the management of plant diseases. This topic has accepted 28 articles on a variety of topics relevant to plant pathogens in agricultural and natural settings, including research and review articles.

## Detection, characterization, and management of plant pathogenic fungi and oomycetes

Fungi and oomycetes are two of the most prevalent pathogen groups that cause diseases in plants worldwide. These pathogens cause significant losses in crop yields, both in terms of quantity and quality, which is a considerable economic burden for the world’s agriculture sector. In order to effectively control fungal and oomycete-caused diseases, pathogen inocula in natural biosystems and agricultural fields need to be accurately and timely diagnosed and characterized (Wang et al., 2020). In both single- and co-infection cases, molecular methods work well when used with conventional approaches to identify symptomatic and asymptomatic diseases caused by both fungal and oomycetes pathogens (Hariharan and Prasannath, 2021). There is still much to be accomplished in the development and implementation of techniques for the diagnosis and characterization of phytopathogenic fungal and oomycete diseases, despite the recent significant expansion of molecular approaches. This section discusses recent developments in the enhancement and utilization of various approaches for the identification, characterization, and control of common and newly emerging plant pathogenic fungi and oomycetes.

Fungi affect the physiological processes of plants and cause a range of diseases. Xing et al. reported that *Botryosphaeria dothidea* causes stem canker and hinders photosynthesis in poplar. They showed that *B. dothidea* inhibits stomatal opening and hampers light energy conversion, electron transport, and light energy usage by poplar leaves. Brauna-Morževska et al. investigated the pathogenicity of *Botrytis* fungal species isolated from various legumes, exhibiting gray mold and chocolate spot diseas. Pathogenicity on tested legume crops was significantly affected by fungal species and isolates. The information indicates that the host range of these *Botrytis* species is broad, and this should be considered when creating succession and crop rotation plans.

Swett et al. reported surveillance for a new I3 resistance gene-breaking race of *Fusarium oxysporum* f. sp. *lycopersici* in California processed tomatoes, following the recent widespread adoption of resistant (F3) cultivars. The fungus *F. oxysporum* f. sp. *lycopersici* (Fol) causes fusarium wilt, which drastically decreases tomato production globally and jeopardizes California’s tomato processing sector. The main management strategy is single-gene resistance, although its effectiveness has been diminished because subsequent races emerged in California. In order to address the challenges associated with distinguishing Fol race 3 from Fol race 4, the authors generated a three-F3 cultivar in a planta test. Using the assay, Fol race 3 was found in all alleged resistance-breaking cases; Fol race 4 was not discovered during these preliminary survey attempts. The findings emphasize the requirement for more study into the factors affecting irregular I3 gene expression in Fol race 3 and for the development of rapid approaches for identifying Fol race 4.

Jeon et al. described the use of direct PCR for phylogenetic research of the *F. fujikuroi* species complex isolated from rice seeds in 2023, which speeds up molecular identification of the fungus by avoiding DNA extraction operations. Twenty-eight potential strains of the *F. fujikuroi* species complex, which causes the bakanae disease of rice and mycotoxin pollution, were identified from rice seeds in the Republic of Korea. The results of phylogenetic and toxicological studies revealed that the *F. fujikuroi* strains may be divided into two categories: FB (B14-type) and FB (B20-type). These findings will hasten the molecular identification of fungal pathogens and make fungal diseases easier to effectively manage.

Virulence, genetic diversity, and evolution of *Puccinia triticina*, causing wheat leaf rust in China’s Hebei Province, were examined by Zhang et al. This research evaluated the genomic and virulence diversity of *P. triticina* isolates obtained from Hebei Province and offers a framework for a better comprehension of how changes in *P. triticina’s* genetic diversity and population structure over time in Hebei.

Hernandez et al. reported application of the heat treatment strategy in the field to decrease *Cercospora beticola* survivability in plant remnants and enhance management of Cercospora leaf spots in sugarbeet. According to this investigation, fall heat treatment considerably decreased lesion sporulation and *C. beticola* isolation in at-harvest samples. Heat treatments in the autumn and spring lowered the area of the disease progression curve, which was examined in the season after treatments.

Zhang et al. used aerial multispectral images and machine learning to diagnose well-known tar spot disease in maize (*Zea mays* L.) at different canopy and temporal levels. Data from multispectral imaging were used to calculate epidemiological parameters, including early disease severity and the area under the disease progress curve. The findings also indicated that digital phenotyping technologies might be used to monitor the creation of tar spots when disease severity is only a little greater than one percent (1%) and to evaluate the efficacy of disease management measures in micro-plot settings. More study is needed to expand and validate these strategies over multi-hectare fields.

Inbaia et al. examined the aggressiveness and mycotoxin profile of *F. avenaceum* isolates generating Fusarium seedling blight (FSB) and Fusarium head blight (FHB) in UK malting barley. *F. avenaceum* has been linked to agricultural production and quality losses, as well as the buildup of mycotoxins such as enniatins (ENNs) A, A1, B, and B1. The study revealed that virulent *F. avenaceum* isolates are prolific ENN producers capable of causing severe FSB and FHB. ENN A1 should be investigated further as a possible virulence factor for *F. avenaceum* in cereals.

Larson and Crandall found that the soil fungal microbiome recovered following steam disinfection to control the plant pathogen *F. solani*. According to the findings, *F. solani* has a significant influence on the relative abundance and diversity of the fungal microbiome in soil; however, *Trichoderma harzianum* is unable to minimize the prevalence of *F. solani* in steam-treated soil. The soil fungal populations with the *T. harzianum* microbial addition were similar to the control (just steaming). This data suggests that *T. harzianum* inoculation has no substantial effect on the formation trajectory of the soil fungal microbiome. Other soil additions, such as a mixture of *Trichoderma* spp. or other species, might impede *F. solani* growth and alter the composition and function of the soil microbiome after steaming.

Gajewska et al. showed that the phytopathogenic oomycete *Phytophthora infestans* can handle redundant sources of internal and host-derived reactive nitrogen species (RNS) in research on the oomycete’s metabolic sensors for nitrosative stress resistance. The study of *P. infestans*’ metabolic sensors’ gene expression, protein synthesis, and enzyme activity in response to nitrosative challenge during both *in vitro* growth and host plant colonization revealed that *P. infestans* is resistant to nitrosative challenge and has a powerful RNS removal system. According to the research*, P. infestans* has a complex network of metabolic sensors that manage RNS balance by detoxifying, allowing the oomycete to thrive in a variety of microenvironments.

Khoo and Chong reported broad pathogenicity characteristics and control strategies of the soil-borne fungus *Ganoderma boninense*. The fungus threatens oil palms, causing basal stem rot and a 43% economic loss for an oil palm plantation within six months. Disease control is challenging due to *G. boninense’s* high persistence technique of soil transmission, which necessitates a thorough investigation of the pathogen’s interactions with host plants as well as the processes behind pathogenicity. In this article, authors presented the general traits, the pathogenic mechanisms, and the host’s defense mechanisms of *G. boninense* and reviewed the most innovative and promising disease management approaches with the least detrimental effects on the environment and natural resources.

Valente et al. used a rapid molecular technique to assess the antifungal efficacy of clove essential oil against a damaging disease of wheat, common bunt, caused by fungal species of the genus *Tilletia*. This study developed a TaqMan Real Time PCR-based test for the rapid identification and measurement of *T. laevis* in young wheat seedlings prior to tillering, which was used in conjunction with phenotypic analysis to investigate the conditions that promote pathogen infection and assess the efficacy of a clove oil-based seed dressing for disease management.

According to Wu et al., the global regulator FpLaeB is necessary for the control of growth, development, and virulence in *F. pseudograminearum*, a soil-borne fungus that can cause a severe crown disease in wheat. During infection, secondary metabolites (SMs), particularly deoxynivalenol (DON), are the key virulence agents. For the regulation of the SM in *Aspergillus nidulans*, authors identified the global regulator FpLaeB, an orthologue of LaeB protein functionality. Findings of this study indicate that FpLaeB plays a key role in cell wall formation, development, and maintenance, as well as membrane integrity. FpLaeB is also essential for SMs and complete pathogenicity in *F. pseudograminearum*.

Li et al. described the genetic and metabolic phenomic properties of *Epicoccum latusicollum*, the causative agent of tobacco Epicoccus leaf spot, which causes large losses in tobacco output and quality in southwest China. Using a mix of conventional techniques and the Biolog Phenotype MicroArray approach, researchers examined the organism’s ideal growing circumstances and metabolic adaptability in order to get a deeper understanding of it. According to the study, *E. latusicollum* showed remarkable metabolic diversity, having the capacity to metabolize most of the carbon, nitrogen, sulfur, and phosphorus sources examined. The study offers fresh perspectives on the composition and dynamics of the phyllosphere microbiota and establishes a theoretical framework for the combined management and breeding of tobacco Epicoccus leaf spot resistance.

The effectiveness of biofungicides on the economically damaging disease dollar spots of turfgarsses was documented by Ghimire et al., along with the susceptibility of *Clarireedia* spp. to benzimidazoles and dimethyl inhibitor fungicides. Findings of this study imply that ongoing monitoring is necessary due to *Clarireedia* spp.’s growing resistance to benzimidazoles and dimethyl inhibitors, and that biofungicides have the potential to supplement synthetic fungicides in a way that is both effective and ecologically benign for disease control.

## Detection, characterization, and management of plant pathogenic bacteria

Plant diseases induced by pathogenic bacteria are a serious problem for a wide variety of plant species worldwide. Accurate identification and characterization of bacterial infections are crucial for the management of a variety of plant diseases (Yang et al., 2023). In this editorial part, we summarize the findings and future directions reported in articles submitted on the Research Topic on the identification, characterization, and management of both beneficial and pathogenic bacteria. These studies encompass future directions in the subject of phytopathogenic bacteria. Utilizing cutting-edge technologies like proteomics and genomics, new methods for extremely accurate identification and characterization of the infections can be devised through investigating their genetic and protein markers (Zubair et al., 2022). Large data sets can also be analyzed by machine learning algorithms, which may provide fresh perspectives on the biology of bacteria that are pathogenic or beneficial as well as innovative approaches to management and diagnosis (Wu and Gadsden, 2023). This section may deepen our knowledge of both beneficial and pathogenic bacteria and encourage the creation of better management strategies for diseases carried on by linked bacterial infections.

Jaffar et al. conducted a brief review of the potential of lactic acid bacteria to intervene in plant disease management and promote crop growth in agricultural production. Recent years have seen renewed interest in research into lactic acid bacteria (LAB), which is a type of bacteria that may be useful in fighting plant pathogens. LAB is increasingly accepted as an option due to its high level of biosecurity and the techniques used to encourage plant growth. This study reviews what is currently known about the antagonistic and phytostimulant properties of LAB, its mode of action, and the benefits and limitations of LAB as a biological control agent to maintain crop productivity.

Kitazawa et al. described random mutagenesis-based screening of the interface of phylogen, a bacterial phylody-inducing effector, to investigate interactions with plant MADS-box proteins. Plant MADS-box transcription factors (MTFs) are a phylody-inducing effector engaged in phylogeny; as a result, the MTF is broken down by the proteasome. The authors discovered two distinct residues where the phylogeny’s affinity for MTF was increased by mutations. Together with all other known interacting residues, these residues are clustered together on the surface of the protein structure of the tree, indicating that they make up the contact interface. Collabfold in silico structural simulations of protein complexes suggest that Phylogen may interact with the K domain of MTF through a possible interface. Their research advances our knowledge of the relationships that exist between phylogeny and MTF.

Through field study in different potato-growing districts of China, Han et al. found and identified the opportunistic pathogen *Pectobacterium polonicum* together with other pectinolytic bacteria that cause potato blackleg or aerial stem rot. These findings of this study imply that the water-associated Pectobacterium species *P. polonicum may* be the first to induce blackleg in a field. *P. polonicum* BY21311 demonstrated a lesser capacity to macerate potato tubers than *P. atrosepticum*, *P. brasiliense*, *P. versatile*, and *P. parvum* despite being less tuber-virulent than the type strain DPMP315T. The genome contents of the two P*. polonicum* isolates appear to differ considerably, and strain-specific genes implicated in a range of activities, including substrate translocation, T4SS, and T6SS, differ between isolates BY21311 and DPMP315T.

According to Wang et al., *Pseudomonas forestsoilum* sp. nov. and *P. tohonis* neutralize 3-hydroxypalmitic acid methyl ester for biological control of bacterial wilt. The establishment of the *Casuarina equisetifolia* protected forest is most threatened by bacterial wilt, which is driven by *Ralstonia solanacearum* and is the world’s second-most important bacterial disease of plants. 3-OH PAME, a crucial quorum sensing (QS) indicator that controls the activity of virulence genes in *R. solanacearum*, is an important focus for disease prevention and treatment. The results of this work offer important information for the identification of downstream quenching genes, the discovery and development of quenching enzymes for disease management, and prospective resources for the prevention and treatment of bacterial wilt brought on by *R. solanacearum*.

Ma et al. investigated the rhizosphere bacteriome in connection to the biological treatment of tobacco black shank disease. The researchers investigated how the soil microbial community influenced black shank disease by examining the diversity and structure of bacterial communities in various rhizosphere soil samples from healthy tobacco, tobacco with black shank symptoms, and tobacco treated with the biocontrol agent *Bacillus velezensis*. Findings from this study increase our understanding of plant-microorganism interactions and how to use biocontrol agents to promote plant fitness.

According to Montesinos et al., eucalyptus oil’s bactericidal and plant defense-inducing properties lessen the severity of *Xylella fastidiosa* infections on almond trees. Using growth inhibition and contact tests, the efficacy of eucalyptus essential oil against eleven strains belonging to six species of plant pathogenic bacteria was investigated. Dependent on the technique employed (endotherapy/soil drenching, preventive/curative), almond plants treated using either method and then infected with *X. fastidiosa* exhibited a considerable decrease in both the disease severity and the levels of the pathogen. Several genes associated with plant defense were elevated in expression in almond plants treated with endotherapy. It was determined that the antibacterial and plant defense-inducing properties of eucalyptus oil treatments were responsible for the decrease in infections.

## Detection, characterization, and management of plant viruses

Plant viral infections provide a substantial challenge to the global agriculture industry (Nicaise, 2014). For the purpose of preventing the spread of disease and developing effective management strategies for both host plants and carrier vectors, plant virus identification as well as prompt detection are crucial (Rubio et al., 2020). Conventional diagnostic approaches, which rely on biological and physical properties of the virus’s size, form, and relationship to its host and/or vector, are too time-consuming and arduous for large-scale testing (Cassedy et al., 2021). Plant virus detection assays that rely on viral protein have been routinely employed for routine and extensive testing throughout the past few decades. When it comes to a quick and accurate diagnosis of plant viral infections, nucleic acid-based techniques outperform serological methods in terms of adaptability, sensitivity, and specificity (Mehetre et al., 2021). This section discusses recent advancements in the field of plant virus detection, characterization, and management. These include field-proven methods and state-of-the-art technologies, such as next-generation sequencing (NGS), that are well-suited for the identification of recently discovered plant viruses. These diagnostic techniques make significant contributions to global crop protection and sustainable agriculture by aiding in plant virus disease prevention and control strategies, characterization investigations, surveillance of diseases research, epidemiological research, plant quarantine, germplasm evaluation, seed certification, and breeding programs.

Nancarrow et al. reported that the symptomless turnip yellows virus (TuYV), which is continually spread by aphids, reduced grain yield in lentils and field peas during three-year field research in south-eastern Australia. Although there is evidence that TuYV infection reduces canola grain production, there is less evidence that it reduces pulse grain yield. The impacts of TuYV infection on lentil and field pea grain production were assessed in this study over three consecutive years (2018–2020) under diverse climatic circumstances. The study revealed that it is critical to screen for viruses rather than relying solely on the presence of visible symptoms in lentils and field peas since the absence of apparent symptoms has significant implications for crop health evaluations.

Cotton leaf curl Multan virus (CLCuMuV), according to Farooq et al., inhibits the innate antiviral immunity of the whitefly (*Bemisia tabaci*) vector in order to enhance the acquisition of cryptic species-dependent viruses. The molecular processes underlying the intricate interactions between begomovirus and whiteflies, which enhance viral transmission, are explored in this study. Researchers previously discovered that while whitefly Asia II 7 cryptic species may easily transmit CLCuMuV, MEAM1 cryptic species is a poor carrier and ineffective vector of CLCuMuV. This study discovered increased virus accumulation in its carrier *B. tabaci* Asia II 7 by selectively targeting and inhibiting the transcription of various antiviral immune responses, including the BTB/POZ gene, which is associated with innate immunity.

Brome mosaic virus was discovered in Kansas wheat co-infected with other common wheat viruses, according to Ranabhat et al. Wheat fields in Kansas were monitored for three years (2019–2021) to employ nanopore sequencing to discover components of natural field virus populations. This study revealed that BMV in Kansas viral populations has made plant breeding significantly more challenging. It is vital to have improved technologies to monitor, detect, and identify changes within BMV.

Wang et al. reported on the genetic variation, phylogeography, and comparability of the alfalfa mosaic virus in China with diverse regional epidemics based on the cp gene. The research reported a protracted, detailed assessment of genetic diversity in AMV populations from China as well as a comparison of the genetics of AMV populations from China, Iran, and Spain. All three countries have similar rates of molecular evolution. Iran had the quickest growth and highest incidence of epidemics, according to the predicted epidemic exponential population size and growth rate, followed by Spain and China.

## Detection, characterization, and management of plant parasitic nematodes

Plant parasitic nematodes cause a wide range of diseases that have a severe impact on agricultural crops, lowering yield and quality and generating significant economic loss (Bernard et al., 2017). Effective nematode management in crop fields requires timely and accurate diagnosis and characterization (Carneiro et al., 2017). Parasitic nematodes frequently exhibit interspecific overlays in addition to significant intraspecific morphological variances, making diagnosis solely using morphological characteristics challenging. On the other hand, molecular techniques are being established, with different levels of accomplishment, to enhance morphology-based approaches and solve current obstacles. A large number of parasitic nematode species have been detected utilizing biochemical and molecular techniques. Recent molecular techniques have been proposed for diagnosing nematodes in the field (Carneiro et al., 2017). These technologies are considered useful since they are quick, accurate, and inexpensive; nevertheless, integrated diagnostics, which combines remote sensing and molecular techniques, is more appropriate for the field (Shao et al., 2023). In this section, we discuss recent research advances as well as the current state of parasitic nematode species detection, characterization, and management measures, with the goal of improving long-term nematode prevention and control in agricultural settings.

Gattoni et al. examined the treatment of *Meloidogyne incognita* by *Bacillus* spp. in 2023 using split root tests, RT-qPCR, and qPCR to determine the mechanism of action of two *Bacillus* species that can suppress *M. incognita* population density in cotton. The greenhouse in planta assay demonstrated that *B. amyloliquefaciens* QST713 and *B. firmus* I-1582, like the chemical standard fluopyram, could control *M. incognita*. According to an *in vitro* study, *B. firmus* I-1582 and its isolated metabolites are capable of directly controlling *M. incognita* second-stage juveniles by increasing the death rate by over 75%. The identification of two bacteria that work via systemic resistance in this study will assist in the adoption of these two species in an integrated management system.

Anderson and Gleason reported a molecular beacon real-time polymerase chain reaction test for detecting *M. chitwoodi*, *M. fallax*, and *M. minor*. *Meloidogyne* spp., often known as root-knot nematodes, are major pests of many important crops across the world. *M. chitwoodi*, *M. fallax*, and *M. minor* harm potatoes in the Northwestern United States of America (USA) because the nematodes can infect tubers, producing galling and a decrease in marketable production. *M. fallax* and *M. minor* are physically similar to *M. chitwoodi*. The newly developed molecular beacon real-time PCR approach can correctly identify *M. chitwoodi*, *M. fallax*, or *M. minor* from crude nematode samples.

Venbrux et al. discussed current and upcoming advances in plant pathogen detection systems. Their operating principles are presented, followed by a review of the primary benefits and drawbacks, as well as instances of their application in plant pathogen detection.

Based on the facts presented above, we can generalize that plants in agricultural and natural systems continually come into contact to and interact with a wide range of pathogens; thus, new fundamental as well as translational studies are required for establishing effective and long-term disease control actions. These research fields include understanding the molecular mechanisms underlying plant diseases, as well as their ecology and epidemiology in the environment. Development of disease-resistant plants, identification of hitherto unidentified pathogens, and identification of novel genes and molecular mechanisms underlying plant-pathogen interactions are examples of notable advances in research in the field of plant pathology (Datta et al., 2023). Recent molecular tools like long-read sequencing technologies are revolutionizing the way we identify and characterize plant pathogens and diseases, as well as how we develop new plant varieties and cultivars (Hamim et al., 2022a). These tools are also changing the way we detect and characterize plant pathogens and pests within plants. It has been possible to develop genetically modified plants that exhibit durable resistance against diseases through molecular research on plants and environmental pathogens (Hamim et al., 2018). The use of genetically modified crops remains debatable due to concerns about possible adverse effects on human health and the environment. For controlling pathogens in the field, sustainable solutions like the employment of hostile microorganisms or other biological control agents may nevertheless be available (Hamim et al., 2022b). Many mycoviruses, for example, have been found to be effective biocontrol agents for plant diseases. These techniques are beneficial to both human health and the environment, but their effectiveness may be limited by the pathogen populations, the environment, and the availability of suitable biological control agents. Furthermore, the implementation of cultural practices including crop rotation, intercropping, and the adoption of resistant varieties of plants are effective strategies for the long-term management of pests and diseases. However, the use of integrated techniques and the establishment of sustainable ways for managing plant diseases require ongoing research into the detection, characterization, and management of plant pathogens.

Finally, this Research Topic offers state-of-the-art methods for detecting, comprehending, and controlling plant pathogens in both agricultural systems and natural habitats. To all the authors and reviewers whose exceptional work made it possible for this Research Topic to be published, we would like to use this opportunity to express our gratitude. We believe that this compilation will increase public knowledge of the significance of plant diseases, enhance monitoring of agricultural and natural systems, enable potential control of emerging plant diseases, and halt epidemics in agricultural crops.

## Author contributions

IH: Conceptualization, Data curation, Formal analysis, Investigation, Methodology, Project administration, Resources, Software, Supervision, Validation, Visualization, Writing – original draft, Writing – review & editing. BS: Conceptualization, Supervision, Writing – review & editing. YW: Writing – review & editing.
